# Mud Banks along the southwest coast of India are not too muddy for plankton

**DOI:** 10.1038/s41598-018-20667-9

**Published:** 2018-02-07

**Authors:** R. Jyothibabu, K. K. Balachandran, L. Jagadeesan, C. Karnan, N. Arunpandi, S. W. A. Naqvi, R. S. Pandiyarajan

**Affiliations:** 1 CSIR-National Institute of Oceanography, Regional Centre, Kochi, India; 2 CSIR-National Institute of Oceanography, Dona Paula, Goa, India

## Abstract

Considering Alappuzha Mud Bank in the southern Kerala coast as a typical case of biologically productive Mud Banks that form along the southwest coast of India during the Southwest Monsoon (June - September), the present study addresses several pertinent missing links between the physical environment in Mud Banks and their influence on plankton stock. This study showed that very strong coastal upwelling prevails in the entire study domain during the Southwest Monsoon, which manifests itself in the form of significantly cool, hypoxic and nitrate-rich waters surfacing near the coast. The upwelled water persisting throughout the Southwest Monsoon period was found to have fuelled the exceptionally high phytoplankton stock in the entire study area, including the Mud Bank region. Having accepted that Mud Banks are special because of the calm sea surface conditions and relatively high turbidity level in the water column around them, the present study showed that except at points close to the sea bottom, turbidity level in the Alappuzha Mud Bank was below the critical level to inhibit the plankton stock. The suspended sediments that form in the Mud Bank occasionally could be attributed to the disturbance of the bottom fluid muddy layer and their vertical spurts.

## Introduction

Mud Bank (Chakara) is a unique and well-known littoral feature that occurs in certain fragmented sections along the Southwest Indian (Kerala) coastline during the Southwest Monsoon (June–September) period^1–4^. Historically, Mud Banks along the Southwest coast of India have been described as semi-circular patches of calm, highly turbid water with a heavy load of suspended sediment that exists within the 10m isobaths^1–5^. When a Mud Bank is formed, the high levels of suspended sediments, especially in the bottom waters (fluid muddy layer), dissipate the energy of the incident waves (wave damping) and create a localised calm environment conducive for fishing activities even as the rest of the region is exposed to the fury of heavy monsoonal waves^3–8^ (Supplementary Figures S1 and S2). From a biological perspective, Mud Banks along the Kerala coast embody calm littoral waters with a high stock of plankton and fishes^1,5–7^. Out of several Mud Banks that occur along the Kerala coast, Alappuzha Mud Bank has especial importance due to (a) its consistent occurrence every year^4,8^ and (b) the large fisheries associated with it^6–9^. Here, in this paper, we consider Alappuzha Mud Bank as a representative case of the several biologically productive Mud Banks that occur along the Kerala coast. It is significant to recall here that even with a dozen hypotheses in place to explain the causative mechanism of Alappuzha Mud Bank, a fool-proof reasoning is yet to emerge^2,3,5,8^.

**Figure 1:**
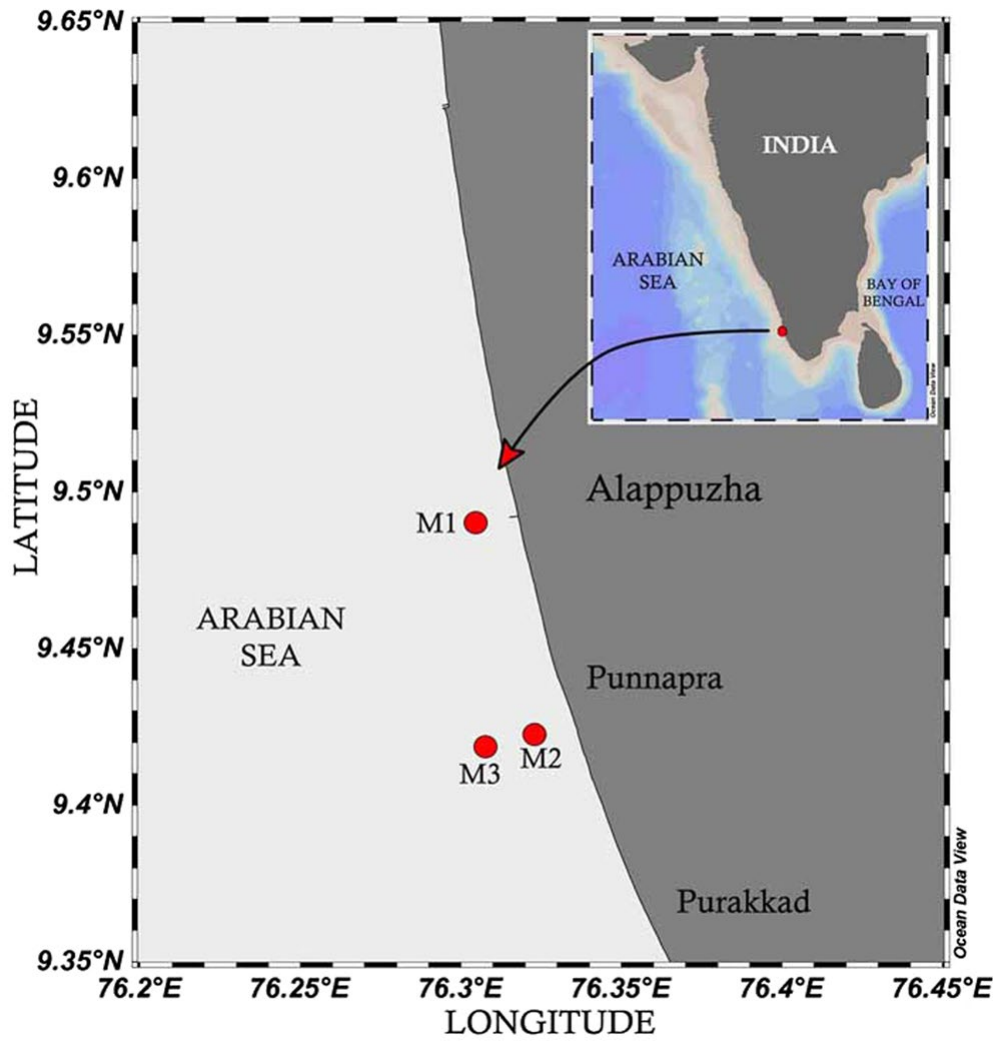
Study area and sampling locations. M2 represent the Mud Bank region and M1 a reference location at the same depth contour and 10 km far from the Mud Bank region. M3 was located beyond the offshore boundary of the Mud Bank, perpendicular to location M2. Weekly/biweekly time series sampling were carried out from all three locations (M1, M2 and M3) from the Pre-Monsoon (March) to the Late-Monsoon (September) in 2014. The map was created with the tool Ocean Data View (version 4.7.10) following the attribution guidelines (Schlitzer, R., Ocean Data View, odv.awi.de, 2017).

It is thought-provoking to observe that some of the earlier studies describing the biological manifestations of Alappuzha Mud Bank have challenged our fundamental scientific reasoning^4,6,7,9,10^. On the one hand, some of these studies describe Mud Banks as highly turbid environments^3–5^ while on the other, highlight the exceptionally high stock of phytoplankton^9^, zooplankton^10^ and fish associated with it^6,7^. Historical literature on Alappuzha Mud Bank has records of chlorophyll *a* ranging from 10–33 mg m^−3^ and micro-phytoplankton abundance from 250–580 cells ml^−1^ ^9^. The foremost scientific reasoning challenged here is how a highly turbid environment in the Mud Bank can support an exceptionally high level of autotrophic plankton stock. The Photosynthetically Active Radiation (PAR) levels in the water column off Alappuzha Mud Bank are also yet to be analysed. It has been noticed earlier in many studies that nitrate concentration in the water column is significantly high when a Mud Bank is formed^9,10^; however, so far there is little clarity about the source of high nitrate in the region. The very high zooplankton stock existing in a highly turbid mud bank is another arguable point, considering that high suspended sediments usually inhibit crustacean plankton growth^11,12^. This study forms the part of a multidisciplinary field programme ‘Alappuzha Mud Bank Process Studies (AMPS)’ executed in 2014 by CSIR-National institute of Oceanography, India, aiming to study various aspects of Mud Banks (Fig. 1). Here, we presented the following aspects of the Alappuzha Mud Bank to comprehend conclusively the actual biophysical coupling of plankton in the region (a) nutrients, turbidity and PAR levels in the water column (b) the phytoplankton and zooplankton stock in the region when Mud Bank is active (c) the oceanographic mechanism involved in the sustenance of high plankton stock in the Mud Bank region, and (d) turbidity in the Mud Bank and its possible influence on the plankton community.

## Results

### Physicochemical parameters

**Figure 2:**
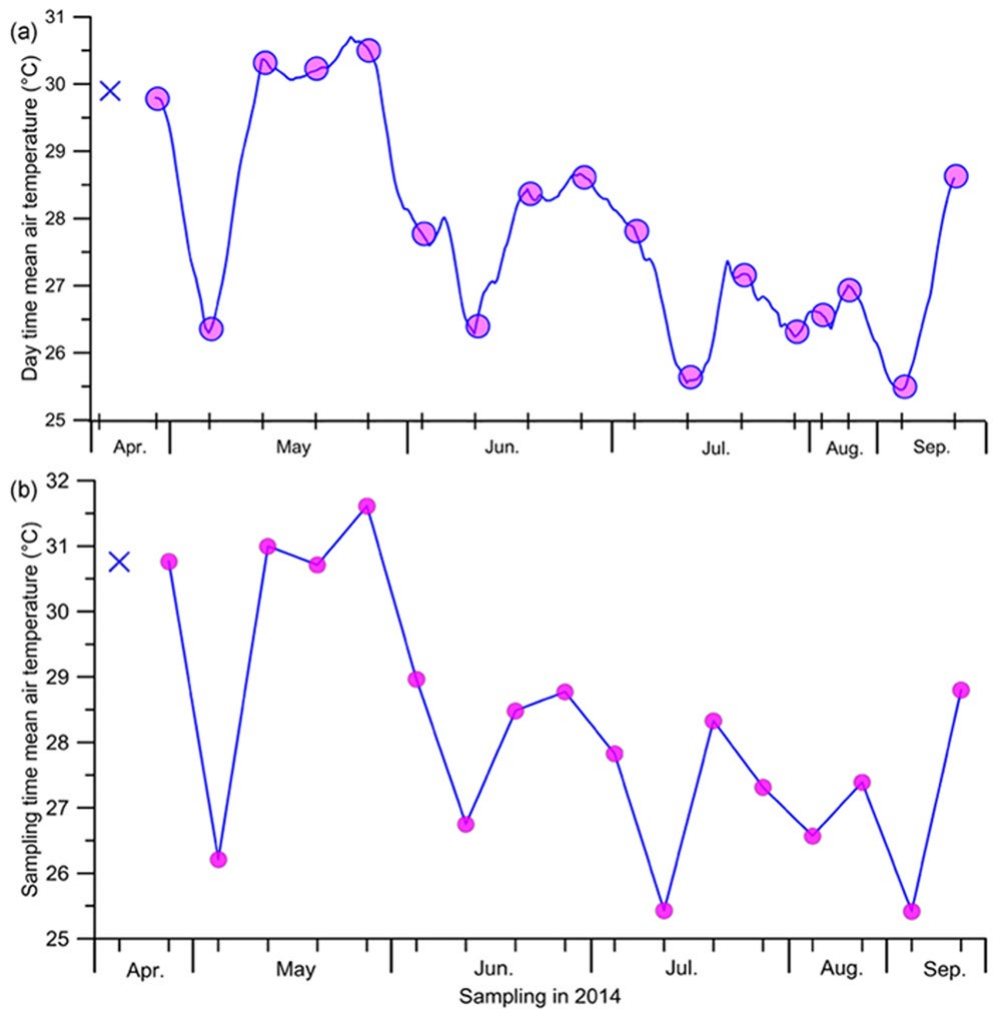
Temporal variation in air temperature. (**a**) Day time (06.00 to 18.00 hrs) mean air temperature and (**b**) sampling time (09.00 to 14.00 hrs) mean air temperature. Air was the warmest during the Pre-Monsoon with an exception during the first week of May when there was a drastic drop due to heavy rainfall associated with a low pressure event. X mark indicates no data.

**Figure 3:**
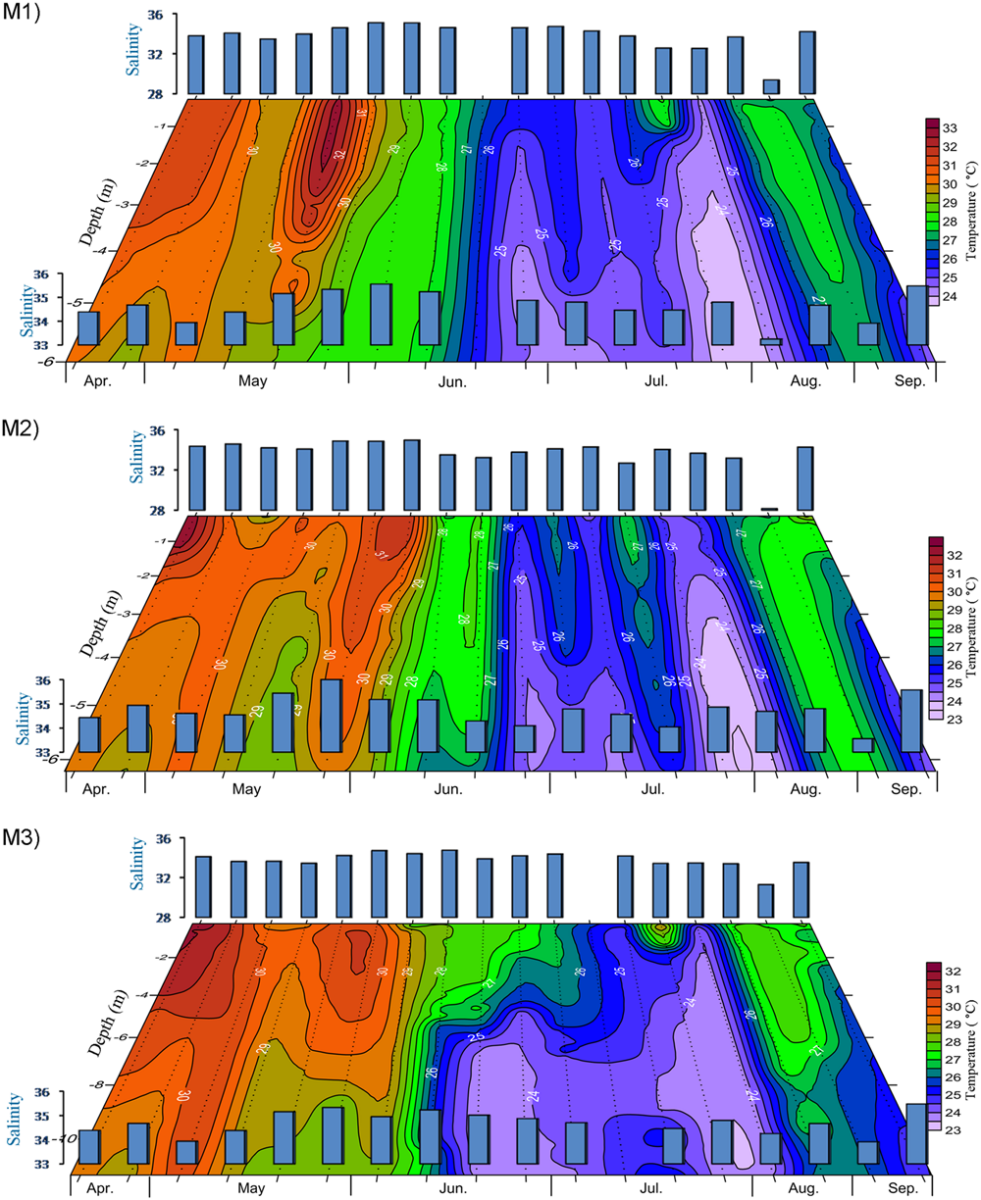
Temporal variations of temperature and salinity at M1, M2 and M3. The colour contour represents the vertical distribution of temperature and blue bars represent the salinity at the surface and subsurface waters. The salinity scales given for the surface and subsurface waters are different.

Air temperature in the study area was observed to be usually high during the Pre-Monsoon; it decreased during the Early- Monsoon and then increased by the Late-Monsoon (Fig. 2). Time series of vertical temperature distribution in the water column also showed a remarkable drop in temperature during the Southwest Monsoon period (Fig. 2), which was more pronounced in the subsurface waters as compared to the surface. The water column was vertically stratified during most part of the Early- and Peak-Monsoon period, with a clear gradient in temperature between the surface and subsurface waters; the surface water was always warmer than the subsurface waters (Fig. 3). The remarkably cooler water in the subsurface as compared to the surface water and air during the Southwest Monsoon period was indicative of the coastal upwelling that prevailed in the region, bringing cool, deeper oceanic waters towards the coast. Salinity in the surface and sub-surface waters was generally high in the study area (>32), and its temporal evolution did not show any clear pattern except a noticeable drop in the surface waters during the Late - Monsoon (Table 1). Lack of any significant change in the surface salinity during the Southwest Monsoon indicated the absence of major freshwater influx in the vicinity of the present study region.

**Table 1:** Physico-chemical and biological parameters at the surface and subsurface (in brackets) waters in M1, M2 and M3.

Stn.	Parameters	Pre-Monsoon	Early-Monsoon	Peak-Monsoon	Late-Monsoon
M1	Temperature (°C)	30.8 ± 1.2 (29.5 ± 0.8)	27.5 ± 1.5 (26.8 ± 2.0)	26.3 ± 1.3 (24.5 ± 0.7)	26.5 ± 1.4 (25.8 ± 1.4)
	Salinity	34.3 ± 0.6 (34.7 ± 0.4)	34.7 ± 0.3 (35.0 ± 0.4)	34.2 ± 0.4 (34.5 ± 0.3)	32.8 ± 2.5 (34.4 ± 0.8)
	Turbidity (NTU)	1.7 ± 0.4 (2.4 ± 0.2)	3.7 ± 0.6 (6.7 ± 2.2)	5.76 ± 0.7 (11.6 ± 0.7)	2.5 ± 1.1 (6.3 ± 0.9)
	PAR (μE m^−2^ S^−1^)	481 ± 404 (15.1 ± 15.7)	522 ± 477 (3.7 ± 5.4)	218 ± 147 (1.8 ± 1.4)	612± 744 (30.3 ± 34.6)
	DO (µM)	190 ± 21 (154 ± 27)	172 ± 69 (67 ± 52)	179 ± 38 (32 ± 26)	192 ± 21 (76 ± 59)
	Nitrate (µM)	0.7 ± 0.9 (1.6 ± 1.8)	1.2 ± 1.0 (3.7 ± 2.7)	3.4 ± 2.1 (6.0 ± 3.3)	1.4 ± 0.7 (3.5 ± 2.8)
	Phosphate (µM)	0.6 ± 0.3 (0.7 ± 0.3)	0.7 ± 0.4 (0.8 ± 0.4)	1.2 ± 0.5 (1.6 ± 0.4)	0.9 ± 0.4 (0.9 ± 0.4)
	Chl. *a* (mg.m^−3^)	1.8 ± 0.9 (1.8 ± 1.6)	5.0 ± 2.6 (2.4 ± 1.0)	9.3 ± 2.4 (5.2 ± 1.1)	3.2 ± 1.8 (3.3 ± 0.5)
	Zoo. Density (No.m^−3^)	3666 ± 2087	1087 ± 199	8401 ± 4995	8082 ± 2478
	Cope. Density (No.m^−3^)	3229 ± 1994	1065 ± 188	8341 ± 2490	5791 ± 1592
M2	Temperature (°C)	30.4 ± 1.1 (29.5 ± 0.6)	27.6 ± 1.6 (26.9 ± 1.8)	25.8 ± 1.4 (24.8 ± 1.0)	27.1 ± 1.6 (25.9 ± 1.7)
	Salinity	34.7 ± 0.2 (34.7± 0.5)	33.9 ± 0.8 (34.6 ± 0.7)	33.9 ± 0.9 (34.6 ± 0.3)	32.9 ± 3.5 (34.1 ± 1.9)
	Turbidity (NTU)	1.7 ± 0.3 (3.2 ± 0.5)	5.4 ± 0.8 (8.2± 2.6)	6.7 ± 1.9 (12.9 ± 0.6)	3.6 ± 2.0 (6.0 ± 1.7)
	PAR (μE m^−2^ S^−1^)	446 ± 361 (24 ± 33)	609 ± 396 (16 ± 27)	206 ± 119 (3 ± 5)	759 ± 553 (31 ± 27)
	DO(µM)	175 ± 25 (128 ± 40)	148 ± 31 (70 ± 18)	200 ± 51 (34 ± 30)	222 ± 34 (120 ± 76)
	Nitrate (µM)	0.7 ± 0.7 (1.9 ± 1.0)	4.2 ± 3.0 (5.6 ± 4.3)	3.3 ± 3.2 (5.9 ± 4.4)	1.2 ± 1.0 (3.9 ± 1.9)
	Phosphate (µM)	0.5 ± 0.1 (0.7 ± 0.3)	0.7 ± 0.2 (1.1 ± 0.4)	1.2 ± 0.4 (1.6 ± 0.3)	0.8 ± 0.5 (0.9 ± 0.5)
	Chl. *a* (mg.m^−3^)	2.7 ± 1.8 (2.1 ± 2.2)	4.5 ± 2.9 (1.7 ± 0.7)	10.7 ± 7.8 (5.7 ± 1.3)	5.0 ± 4.5 (3.9 ± 1.8)
	Zoo. Density (No.m^−3^)	3628 ± 529	2815 ± 1740	8053 ± 4511	6509 ± 3119
	Cop. Density (No.m^−3^)	3328 ± 1245	2403 ± 1347	7595 ± 4410	5554 ± 2366
M3	Temperature (°C)	30.0 ± 1.4 (29.0 ± 0.8)	27.5 ± 0.7 (24.3 ± 0.9)	26.9 ± 2.1 (24.3 ± 0.8)	26.7 ± 1.6 (25.3 ± 1.2)
	Salinity	34.3 ± 0.7 (34.9 ± 0.4)	34.3 ± 0.4 (35.0 ± 0.5)	34.0 ± 0.5 (34.7 ± 0.1)	33.0 ± 1.1 (35.3 ± 0.3)
	Turbidity (NTU)	0.6 ± 0.2 (1.7 ± 0.8)	1.7 ± 0.7 (10.2 ± 0.3)	3.72 ± 1.9 (9.3 ± 0.7)	1.4 ± 1.3 (6.2 ± 0.7)
	PAR (μE m^−2^ S^−1^)	729 ± 615 (69 ± 63)	680 ± 751 (14 ± 24)	315 ± 243 (4 ± 4)	317 ± 172 (4 ± 6)
	DO (µM)	181 ± 10 (147 ± 30)	147 ± 12 (16 ± 11)	226 ± 61 (30 ± 19)	248 ± 74 (42 ± 25)
	Nitrate (µM)	0.4 ± 0.5 (2.3 ± 1.4)	0.5 ± 0.4 (11.3 ± 5.4)	1.7 ± 1.5 (11.1 ± 2.1)	1.3 ± 1.1 (11.4± 8.5)
	Phosphate (µM)	0.6 ± 0.6 (0.9 ± 0.2)	1.1 ± 0.6 (1.7 ± 0.3)	0.9 ± 0.2 (1.8 ± 0.2)	1.0 ± 0.9 (1.3 ± 0.5)
	Chl. *a* (mg.m^−3^)	0.7 ± 0.2 (0.6 ± 0.4)	7.4 ± 6.4 (2.1 ± 1.5)	10.1 ± 7.6 (6.0 ± 3.0)	4.0 ± 3.0 (2.8 ± 2.4)
	Zoo. Density (No.m^−3^)	2432 ± 1727	4680 ± 4625	5242 ± 1724	7420 ± 3872
	Cope. Density (No.m^−3^)	1817 ± 1382	4482 ± 1084	4881 ± 1553	7080 ± 3723

**Figure 4:**
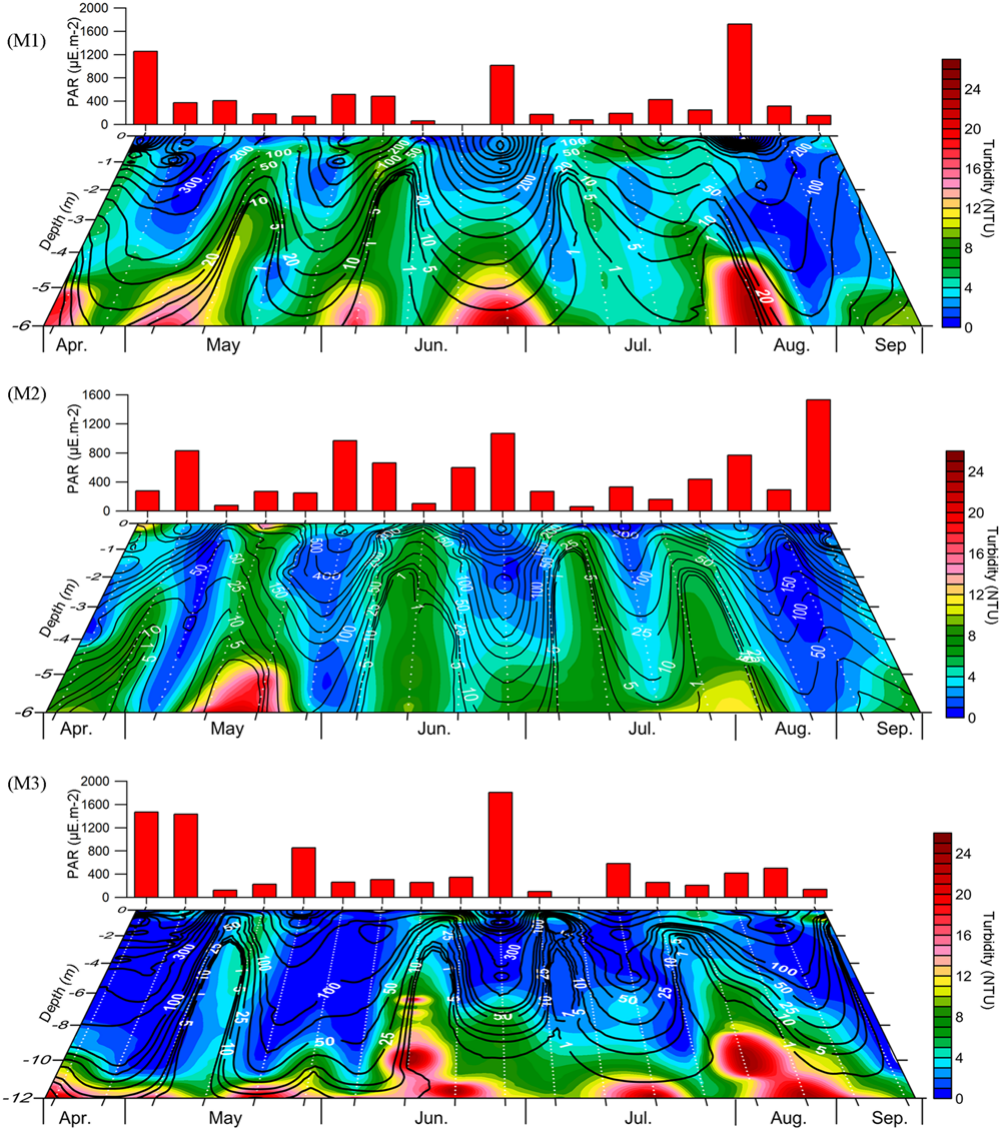
The background colour shading indicates the temporal change in the vertical distribution of turbidity in M1, M2 and M3. The black contours lines with white labels represent the temporal variations in underwater Photo-synthetically Active Radiation (PAR) and the red bars on top of the panels represent PAR at the surface waters (0.5 m depth).

**Figure 5:**
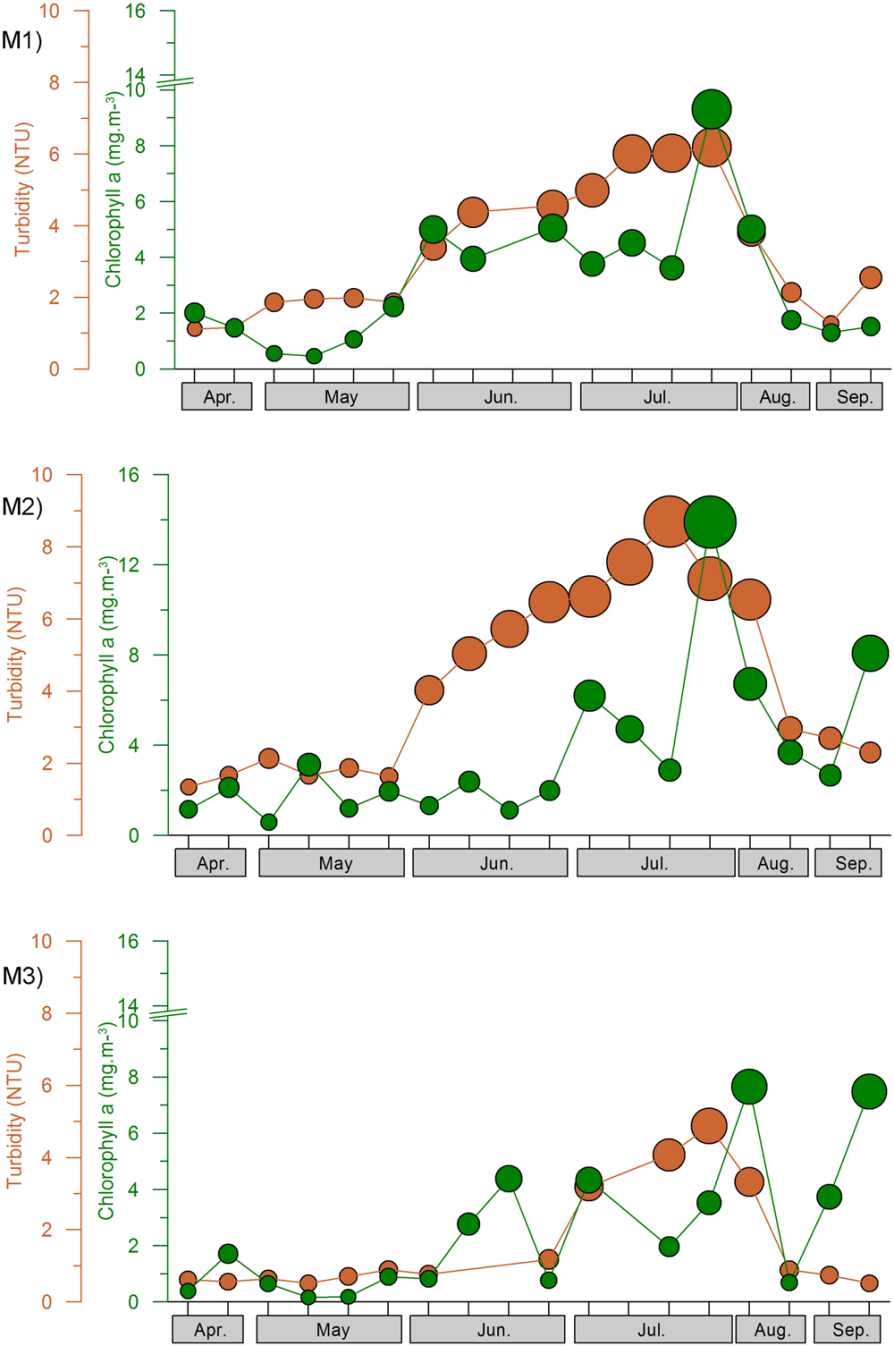
Temporal distribution of turbidity (brown bubbles) and Chlorophyll *a* (Green bubbles) at M1, M2 and M3. The bubble size represents the proportionate values of the specific parameter.

Vertical distribution of turbidity and PAR indicated the signatures of a highly turbid, fluid muddy layer close to the bottom at all the three locations throughout the sampling period (Fig. 4). Intermittently, the bottom fluid muddy layer increased its height and produced upward spurts of turbidity. Such events were more pronounced at the M2 location during the Southwest Monsoon, causing increased turbidity in the entire water column. At other locations, the vertical spurts of sediments were limited mostly to the subsurface waters (Fig. 4) despite the presence of a fluid muddy layer in the bottom. However, on no occasion did the water column remain turbid for longer periods at any of the three locations, even during the Southwest Monsoon. Also, what is clear in the data is the episodic recurrence of a relatively turbid water column at all locations even during the Southwest Monsoon period when a Mud Bank was formed at the M2 location (Figs 4 and 5; Supplementary Figure S2).

Underwater PAR showed a significant negative relationship with turbidity, especially in the sub-surface waters, its noticeable highs and lows following a trend opposite to that of the water column turbidity. The surface PAR presented in Fig. 4 shows several sampling days that were cloudy with less than 100 μE m^−2^ S^−1^ PAR at the surface waters. Some of these sampling days coincided with elevated turbidity levels in the water column during the Southwest Monsoon. Only under such conditions, >1 μE m^−2^ S^−1^ of underwater PAR was restricted to the upper 2–3 metres of the water column, and such occasions were only 3 out of 12 field sampling instances carried out in the study region during the Southwest Monsoon. In all the other sampling sessions in M1 and M2 (9 out of 12) during the Southwest Monsoon, the entire water column showed high availability of PAR (Fig. 4). Location M3 was unique with respect to PAR availability in the sub-surface waters during the Southwest Monsoon, as the vertical spurts of the fluid muddy layer significantly inhibited the same.

Dissolved oxygen concentration in the sub-surface waters at all the three locations showed clear temporal change (Table 1; Supplementary Figure S3). The sub-surface concentration of dissolved oxygen showed a sharp decrease during the Early - Monsoon period. It further decreased during the Peak - Monsoon period, reaching hypoxic levels (Table 1). The nitrate and phosphate concentration showed significantly higher values in the surface and subsurface waters during the entire Southwest Monsoon period as compared to the Pre-Monsoon. Higher concentration of both nutrients (nitrate, phosphate) during the Southwest Monsoon was found in the sub-surface waters as compared to the surface (Table 1; Supplementary Figure S4). Similarly, the concentration of nitrate in the surface waters was found to be higher at the location closer to the coast (M1 and M2) as compared to the one in the offshore region (Table 1); however, these differences were statistically insignificant. Over all, ANOVA results (Supplementary Table S1) showed that the variations in all environmental parameters, except turbidity at the surface waters, between M1, M2 and M3 locations (spatial variations) were insignificant (P > 0.05). Conversely, significant temporal variation (P < 0.05) in environmental parameters, except salinity, was evident in the case of subsurface waters during the sampling period (Supplementary Table S1).

### Plankton response and ecology

**Figure 6:**
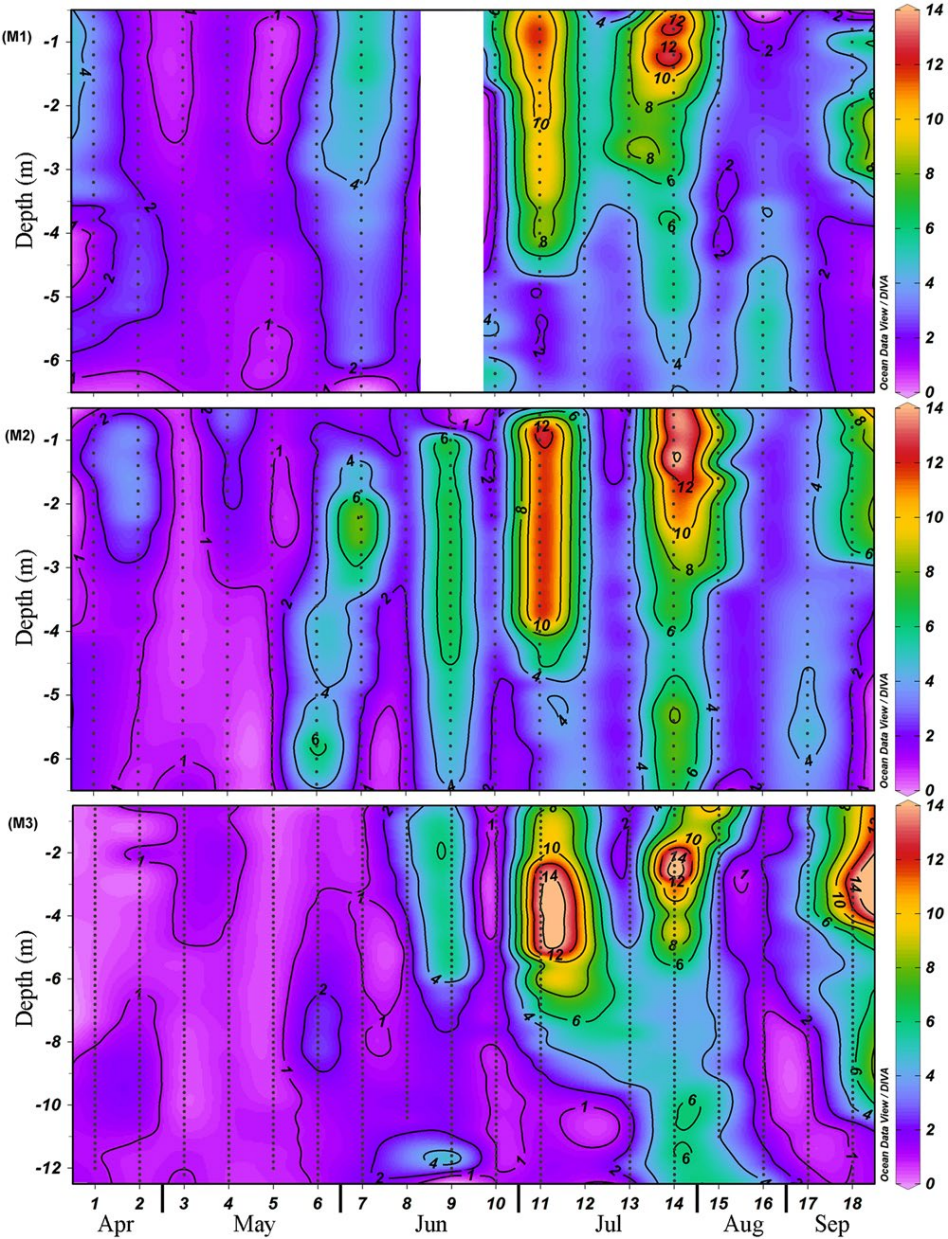
The gradients in the contours and colour shading indicate the temporal evolution in the vertical distribution of chlorophyll *a* at M1, M2 and M3. The time series of the vertical distribution of chlorophyll *a* clearly showed a significant increase in values during the Southwest Monsoon period (June - September) in all three locations associated with coastal upwelling. Also evident that significantly high chlorophyll concentration was present in the subsurface waters during the Southwest Monsoon.

Chlorophyll *a* in the water column showed a low concentration phase during the Pre-Monsoon, whereas a high concentration phase was observed during the Southwest Monsoon (Fig. 5). The increase in chlorophyll *a* during the Southwest Monsoon was exceptional and in most instances, was several folds higher than the Pre-Monsoon levels. Within the Southwest Monsoon period, the highest concentration of chlorophyll *a* in the surface and sub-surface waters of all locations was observed during the Peak - Monsoon period (Table 1). It was also evident in the vertical distribution of chlorophyll *a* that the highest concentration occurred in the subsurface waters between 0.5 to 5 m at the coastal locations (M1 and M2) and 0.5 to 6 m at the offshore location (M3) (Fig. 6). During the Pre-monsoon period, the phytoplankton was dominated by smaller diatoms *Asterionella*, *Odontella*, *Cerataulina*, *Chaetoceros* and *Leptocylindrus*, with a mean cell size usually smaller than 100 µm. On the other hand, the plankton community during the Southwest Monsoon was dominated by diatoms *Fragilaria*, *Coscinodiscus*, *Hemidiscus*, *Guinardia*, *Rhizosolenia*, *Chaetoceros* and *Asterionella* and all of them were significantly larger in size (>100 µm). During the Peak and Late - Monsoon, *Fragilaria* and *Coscinodiscus* formed blooms at all three locations. Detailed information on the phytoplankton size structure, composition and temporal evolution in the present study region has been presented by Karnan *et al*.^13^.

**Figure 7:**
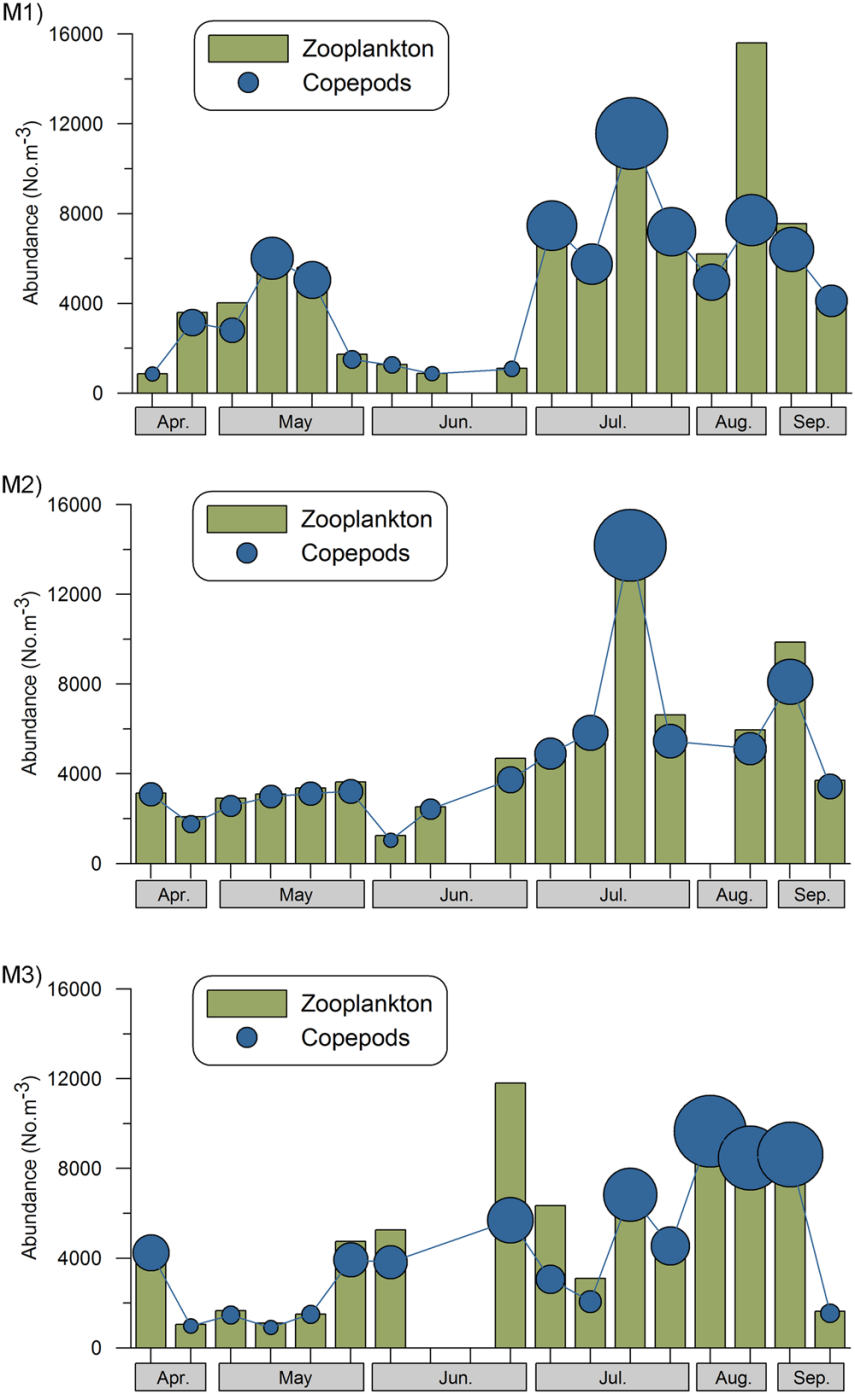
Spatial distribution of meso-zooplankton and copepods abundances at M1, M2 and M3. The bubble size represents the proportionate values of copepod abundance.

The mesozooplankton biomass and abundance showed noticeable temporal fluctuations in the study area (Supplementary Figure S5). At all three locations during the Pre-Monsoon and Early-Monsoon, zooplankton biomass was <1.5 ml.m^−3^, which later increased and became exceptionally high (>10 ml.m^−3^) during the Peak and Late - Monsoon. Zooplankton density in the study region varied temporally from 863 to 15611 ind.m^−3^, with lower values during the Pre-Monsoon compared to Peak and Late -Monsoon periods. 13 taxonomic groups contributed to the zooplankton community in which copepods formed the majority (49–96%). Copepod density was generally high during the Peak and Late-Southwest Monsoon compared to Pre-Monsoon and Early-Monsoon (Fig. 7). The other groups contributing to the mesozooplankton community included chaetognaths, ostracods, hydromedusae, siphonophores, cladocera, lucifer, thaliaceans, fish larvae, appendicularians, mysids and polychaete larvae, but these together contributed only <5% of the total zooplankton abundance. Out of 43 species of copepods identified from the study area, there were 25 Calanoids, 8 Poecilostomatoids, 5 Cyclopoids and 5 Harpacticoids. The copepod abundance was the highest during the Peak- Monsoon as compared to the other periods. During Pre-Monsoon, *Acartia danae*, *Acartia erythraea*, and *Centropages orsini* were dominant at all three locations. During the Early - Monsoon, *Pareucalanus attenuatus*, *Temora turbinata, Acartia erythraea, Acartia danae* and *Oithona similis* were dominant. During the Peak- Southwest Monsoon, the contribution of Cyclopoids was much higher than other families; the contribution of *Oithona similis* alone was 60–80% of the total copepod abundance. During the Late-monsoon, the dominant copepods consisted of *Centropages tenuremis, Pseudodiaptomus serricaudatus, Temora turbinata, Euterpina acutifrons* and *Oithona similis*. The detailed information on zooplankton community composition in the study region have been presented in the research contribution of Jagadeesan *et al*.^14^.

**Figure 8:**
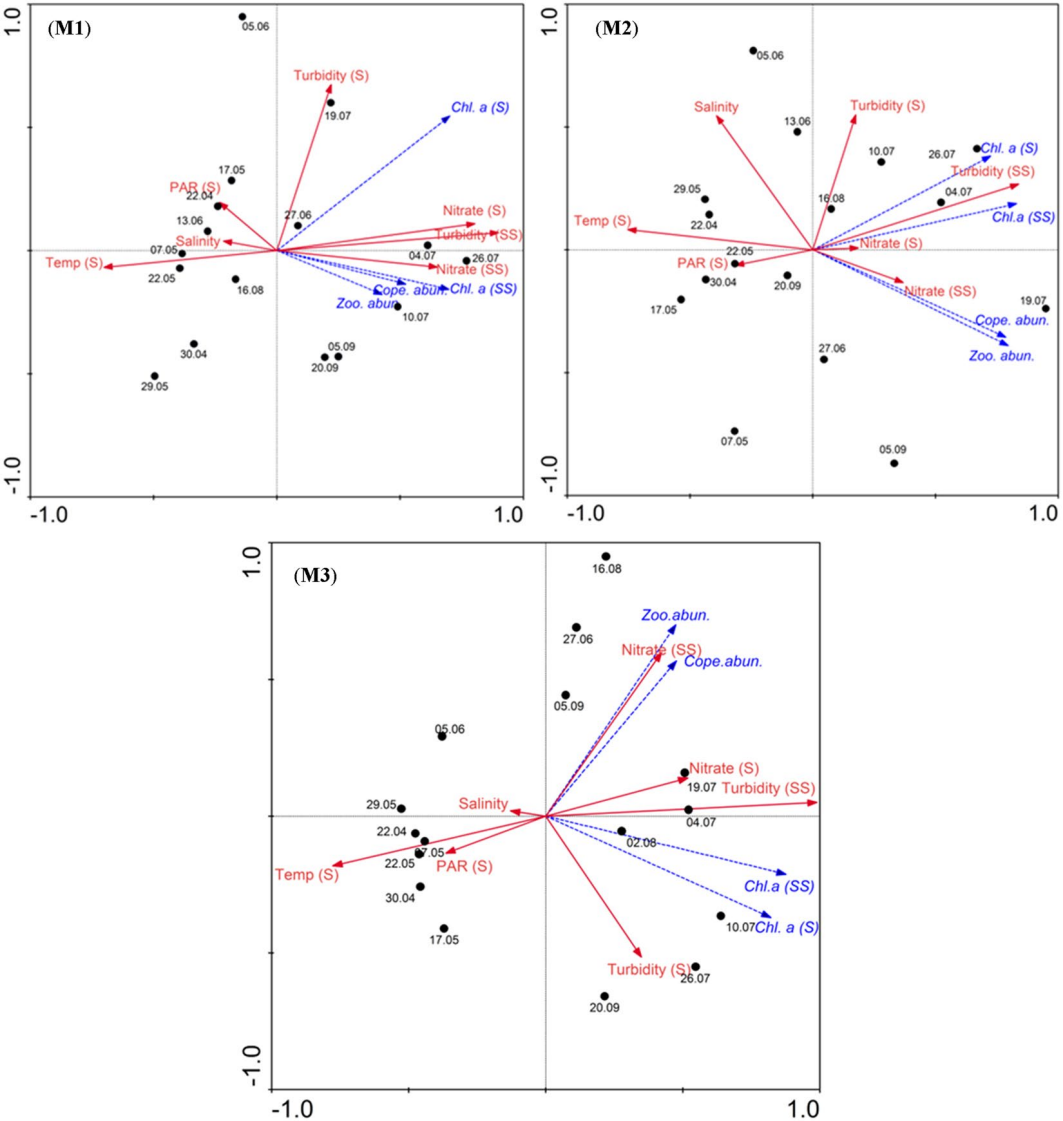
RDA Triplot showing the overall inter-relationship of plankton parameters with other environmental variables during the study period. It showed high values of all biological parameters towards the region (right hand side of the plots) represented by low temperature, high nutrients and turbidity representing the Southwest Monsoon period. Abbreviations: S - Surface, SS - subsurface, Temp. - Temperature, Cope. abun. - Copepod abundance, Zoo. abun. - Zooplankton abundance.

The RDA analysis presented the inter-relationship between the physico-chemical parameters and also the relationship of the plankton components with environmental parameters. In the RDA Triplot, samples were displayed by points, and environmental and biological variables by solid and dotted arrows, respectively (Fig. 8). The Triplot presented the data of all the 18 field sampling sessions carried out during the present study to visualise the distribution of the physico-chemical and biological parameters and their inter-relationships. The physicochemical parameters considered for the present RDA analysis included temperature, salinity, PAR, turbidity and nitrate, whereas, the biological parameters considered were chlorophyll *a*, zooplankton biomass, zooplankton density and copepod density. In the Triplots, temperature, salinity and PAR were oriented on the left-hand side, represented mostly by Pre-Monsoon samples. The turbidity, nitrate and all biological parameters were oriented on the right-hand side, represented mostly by the Southwest Monsoon samples.

## Discussion

That calm sea conditions in the Mud Banks along the Kerala coast occur due to wave damping at their offshore boundary is a well-established feature^1–8^. A snapshot of the calm sea conditions in the Alappuzha Mud Bank region recorded during the present study has been provided here (Supplementary Figures S1, S2 and Supplementary Video SV1). Mallik *et al*.^15^ concluded that the term ‘Mud Bank’ is not really applicable to the feature of calm, relatively turbid waters that form along the Southwest coast of India. In the true sense, a ‘Mud Bank’ is a submerged or partly submerged ridge or relatively flat-topped elevation on the sea floor at shallow depth, which may remain unexposed even at low tide^15,16^. Considering the nature of the sediments suspended in the Mud Banks along the Southwest coast of India, ‘fluid mud’ has been suggested as a more apt term instead of ‘Mud Bank’^15,16^. As detailed in the introduction, the physical mechanism behind the Alappuzha Mud Bank formation is still undergoing scientific revision and is, therefore, truly uncertain at the moment^1–5,8^; on the other hand, its biological manifestations in terms of plankton and fisheries are certain^11,14^. Historical as well as recent studies have confirmed high plankton production in the Alappuzha Mud Bank and restate that a rich fishery is associated with it, usually referred to as ‘Mud Bank fishery’^6,7^. On the other hand, studies on benthos in relation to the sediment characteristics indicate that the Mud Bank bottom sediments are poor in terms of faunal abundance and diversity^17,18^. In the Mud Bank region, the top layer of the bottom sediments occurs in a highly unconsolidated and loose state (fluid muddy layer) when the Mud Bank is active. A conspicuous feature of the Mud Bank region is the loose mud (mostly formed of very fine clay) in suspension over the unconsolidated sediment^17,18^. Therefore, it is quite reasonable to directly link the disturbed bottom sediment and very poor bottom faunal diversity and biomass in the Mud Bank area^17,18^.

The most significant study regarding the biological implications of coastal upwelling along the southwest coast of India was conducted by Banse^19^. Since then several researchers have studied different biological aspects of the coastal upwelling prevailing in this region^20–23^. Research over the last several decades has categorically established that coastal upwelling during the Southwest Monsoon plays a crucial role along the south-western Indian shelf in increasing the plankton production and fish stock^20–23^. What is still unclear is the shoreward extent of the coastal upwelling along the Southwest coast India, as there is a possible capping effect by the lens of freshwater at the surface layers originating from the river influx. Considering the numerous rivers (>50 numbers) along the Southwest cost of India and their maximum influx during the Southwest Monsoon, the general belief is that it can be a combined effect of both coastal upwelling and river influx that eventually supports high biological production in the near-shore waters^20,22–25^.

The most striking hydrographical feature observed in the study area during the Southwest Monsoon was the well-defined signature of coastal upwelling, including a significant drop in temperature and dissolved oxygen in the subsurface waters. This was reflected clearly in the ANOVA results displaying significant temporal variation in temperature (p < 0.01) and dissolved oxygen (p < 0.05) in the subsurface waters at all three locations. Noticeably cooler water in the subsurface layers as compared to the surface, during the Southwest Monsoon, indicated the presence of upwelled waters. Probably, the present study forms one of the most important records from this region depicting upwelled waters reaching so close to the coast. It is pertinent to mention here that a few earlier studies from the southwest coast of India have also showed upwelled waters close to the coast^20,26^ and even it intruding into the estuarine bottom through deep channels in the estuarine inlets during the Southwest Monsoon^26–28^. ANOVA showed that the spatial differences in nutrient concentration between the three locations (M1, M2 and M3) were insignificant (p > 0.05) throughout the study period. At the same time, the temporal variation in nutrient concentration was similar and significant (p < 0.05) at all the three locations. These findings signify the exceptionally high nutrient concentration present in the Alappuzha Mud Bank during the Southwest Monsoon driven primarily by the coastal upwelling phenomenon that occurs at a large spatial scale.

An increase in turbidity and nutrients in the near-shore waters along the southwest coast of India is quite expected during the Southwest Monsoon due to increased river influx and land runoff during the period^23–26^. However, in the present study, the typical Mud Bank signature of suspended sediment-induced dampening of waves and calm sea surface was found only at Location M2 during the Southwest Monsoon. The details of the prevalence of the fluid muddy layer in the bottom waters of the study domain leading to the formation of Mud Bank has been presented recently in another manuscript by Shynu *et al*.^29^. In the present study, chlorophyll *a* concentration was found to be low in the study area during the Pre-Monsoon, which showed a noticeable increase at all locations during the Southwest Monsoon. This would mean that the transformation evident in all the hydrographical data collected from the study domain during the Southwest Monsoon was favourable for an increase in the plankton stock. The Pre-Monsoon in the South-eastern Arabian Sea is a biologically less productive period as compared to the Southwest Monsoon due to the relatively low availability of nutrients^23,25,30^. In other words, the high availability of nutrients in the South-eastern Arabian Sea during the Southwest Monsoon favours significant growth in the phytoplankton community. As a result, most of the diatom blooms along the southwest coast of India occur during the Southwest Monsoon period^9,23,25,30,31^. It is straightforward to consider that optimum photosynthetically active radiation (PAR) and nutrient levels are the two most important factors making a marine environment conducive for the proliferation of phytoplankton^32–34^. Usually, the phytoplankton community in the surface waters of tropical oceans grows under high light conditions with low chlorophyll content^31,32^.

One pertinent question to be analysed here is the nature and level of turbidity in the Alappuzha Mud Bank area during the Southwest Monsoon period. During the period when Mud Bank forms in the study domain, a thick ‘fluid muddy layer’ exists close to the sea bottom^1,3,15^. This loose fluid muddy layer is considered to be crucial for attenuating the wave energy and generating calm sea conditions, which is visually discernible when Mud Banks exist in the region^1,2,5,15^ (Supplementary Figures S1, S2 and Supplementary Video SV1). As evident in Fig. 4 and detailed in the results, the bottom fluid muddy layer found in the study area exhibited an intermittent increase in its height and produced upward spurts of turbidity. The physical mechanisms behind such intermittent events leading to the suspension of bottom sediments have been debated in detail over the years but remain unresolved^3,5,8^. More important here is to note that although a fluid muddy layer was present at the bottom of all the locations studied, the vertical spurt of sediments in these locations were mostly limited to the subsurface waters except in the case of M2 location during the Southwest Monsoon where increased turbidity was evident occasionally in the entire water column (Fig. 4). However, the water column did not remain turbid for long periods of time, even during the Southwest Monsoon period; the observation was only an episodic recurrence of relatively turbid water column even at M2 location when the Mud Bank existed there (Fig. 4). These observations strongly suggested that Alappuzha Mud Bank is not so muddy even during the Southwest Monsoon. Considering the above, the propositions of Mallik *et al*.^15^ seem to be relevant as they considered Kerala ‘Mud Banks’ as ‘fluid muds’ close to the sea floor.

One of the intriguing aspects of the Alappuzha Mud Bank in relation to the biological production was the difficulty in scientifically explaining how such an environment with high suspended sediments could support high phytoplankton and zooplankton production. The level of water column turbidity at M2 when Mud Bank prevailed was apparent in the present study (Fig. 4). Closely associated with the turbidity was the response of underwater PAR, which showed a significant negative relationship with turbidity. During the Southwest Monsoon period, when Mud Bank prevailed at location M2, a few of the sampling days were significantly cloudy with noticeably low surface PAR (<100 μE m^−2^ S^−1^). A few such sampling days coincided with elevated turbidity levels in the water column and only on such conditions, PAR underwater (>1 μE m^−2^ S^−1^) was mostly restricted to the upper 2–3 metres of the water column. In all other sampling cases (9 out of 12) during the Southwest Monsoon, the entire water column showed high PAR availability (Fig. 4). In the case of location M3, the situation was slightly different in the bottom waters during the Southwest Monsoon as the fluid muddy layer close to the bottom was around 3m thick and significantly inhibited the PAR availability in the bottom layers. It is also relevant to consider the fact that heavily cloudy sky conditions are mostly transient features, which usually last only for a few hours, and after the rainfall, PAR availability over the ocean surface increases significantly. An exception to this may occur during unusual occasions such as atmospheric depression events, and in such cases, cloudy conditions may last consistently for a few days.

A significant increase in chlorophyll *a* was evident at all the locations during the Southwest Monsoon compared to that during the Pre-Monsoon. It was also evident during the Southwest Monsoon that the highest concentration of chlorophyll *a* occurred in the subsurface waters between 0.5 to 5m at the coastal locations (M1 and M2) and 0.5 to 6m at the relatively offshore location (M3) (Fig. 6). This essentially confirmed that there was no light inhibition on the phytoplankton stock in the water column during the Southwest Monsoon due to increased turbidity. The phytoplankton community in the study area during the Southwest Monsoon was dominated by larger diatoms *Fragilaria*, *Coscinodiscus*, *Hemidiscus*, *Guinardia*, *Rhizosolenia*, *Chaetoceros* and *Asterionella*. Phytoplankton blooms were evident in the subsurface waters during a few observations throughout the peak and late Southwest Monsoon, which was evident in the trail of the sampling while sailing between the sampling locations (Supplementary Figure S6 and Supplementary Video SV2). Considering the usual timing of the bloom formation of these larger diatoms along the southwest coast of India^9,31^, mostly during the peak and late Southwest Monsoon, it is quite likely that the optimum solar radiation requirements of these larger diatoms could be low. This has to be considered in association with the fact that the subsurface light availability in the near-shore waters along the Southwest coast of India is considerably low during the Southwest Monsoon due to increased water column turbidity from the river influx and also the cloud cover^9,31,33^. Earlier observations from the west coast of India showed that the diatom bloom occurs in the region during the Southwest Monsoon period^9,31,33^. Our very recent experimental data from the west coast of India also showed that the light requirement of large diatoms (*Fragilaria* and *Coscinodiscus*) for their optimum growth is 30–70 μEm^−2^ S^−1^ (Unpublished). These observations convey the fact that the PAR available underwater off Alappuzha Mud Bank does not impart any inhibitory effect on the native phytoplankton community in most parts of the water column (5 m out of 7 m at M1 and M2; 7 m out of 13 m at M3) and, therefore, the region harbours significantly high phytoplankton biomass. This feature was clearly evident in the present RDA analysis, wherein increasing chlorophyll *a* gradients were oriented towards samples with cool temperature, low PAR and high turbidity, all representing the Southwest Monsoon (Mud Bank period) in the study area.

Studies from elsewhere show that the minimum light requirement of phytoplankton growth is quite low. Geider *et al*.^34^ showed that the minimum amount of light required for net growth by phytoplankton *Bacillariophycea* is 0.5 μE m^−2^ s^−1^. In coastal waters of Oregon, Wetz *et al*.^35^ found that net phytoplankton growth required approximately 0.75 mol quanta m^−2^ d^−1^ (8.6 μmole m^−2^ s^−1^). In the North Pacific subtropical gyre, Letelier *et al*.^36^ concluded that the compensation depth corresponded to approximately 0.45 μmole quanta m^−2^ d^−1^ (5.2 μmole m^−2^ s^−1^). All the above suggest that the significant increase in phytoplankton biomass observed in the present study, at all the three locations during the Mud Bank period (Southwest Monsoon), is the result of the low PAR requirements of the phytoplankton community thriving along the southwest coast of India. Based on past studies in turbid aquatic environments, the expected impact of turbidity on zooplankton community has been reviewed by Levin *et al*.^11^. Increasing turbidity has diverse undesirable impacts on the zooplankton community, which includes mechanical interference in the ingestion of food particles^11,37,38^. Sediment brought into the gut reduces the efficiency of food assimilation and makes animals heavier^11,38,39^. Energy is expended both in countering increased sinking rates and in rejection of sediment-laden boluses. These elevated respiratory demands combined with lower food intake and sediment abrasion of exoskeletons may result in poorer animal condition, lowered fecundity, and reduced survivorship^11,37,39^.

It was observed that the overall spatial trend in zooplankton biomass, composition and abundance at all the three locations sampled in the present study area were similar. The zooplankton biomass was found to be low (<1.5 ml.m^−3^) during the Pre-Monsoon and Early-Monsoon compared to the exceptionally high values (>10 ml.m^−3^) during the Peak and Late - Monsoon. This was quite understandable as Pre-Monsoon is a low productive season and during the Early- Monsoon the zooplankton biomass remains low due to the time lag involved in nutrient enrichment; also, subsequent production of phytoplankton and zooplankton stock at all the three locations, the copepod composition and its temporal changes were largely the same. During the Early - Monsoon when the Mud Bank formed at location M2, copepods *Pareucalanus attenuatus*, *Temora turbinata*, *Acartia erythraea*, *Acartia danae* and *Oithona similis* were dominant at all the three locations. Among these species of copepods, *Temora turbinata* is considered as an indicator of the coastal upwelling phenomenon (Supplementary Figure S7). During the Peak- Monsoon when Mud Bank prevailed at M2, cyclopoid copepods were predominant at all the three locations. The above observations suggest that the environmental condition at the Mud Bank location (M2) was not very different from the other two locations (M1 and M3) for zooplankton stock, signifying the fact that the level of turbidity in the Mud Bank region does not introduce a significant level of inhibition on zooplankton growth. More importantly, it is relevant to note that the turbidity level in the water column in the Mud Bank during the Southwest Monsoon, even during the turbid days, was <10NTU, which is quite low. Also, such level of turbidity is usual in the adjacent estuarine waters (Cochin backwaters) even during the Pre - Monsoon, when the zooplankton community biomass and abundance remain the seasonal highest. All the above observations explain how high zooplankton stock is sustained in the Alappuzha Mud Bank without contradicting the basic scientific reasoning that high suspended sediments inhibit crustacean plankton growth^11,37–39^. The present study introduced the idea that Alappuzha Mud Banks are unique because of the calm sea surface conditions and relatively lower turbidity in the water column, and the exceptional turbidity here is restricted only to the fluid muddy layer close to the bottom. Also, this study presented the view that the plankton stock in the Alappuzha Mud Bank during the Southwest Monsoon is mainly governed and fuelled by the intense coastal upwelling. This study concludes that Alappuzha Mud Bank is not too muddy for a flourishing plankton community along the southwest coast of India during the Southwest Monsoon.

## Sampling and Methods

Three locations (M1, M2 and M3) were selected south of Alappuzha, along the Southwest (Kerala) coast of India (Fig. 1). M2 (at 7 m depth) represented the region where Mud Bank and coastal upwelling co-occur during the Southwest Monsoon. Locations M1 and M3 represented references, the former in the shallow waters (7 m) and the latter in the offshore region (13 m depth) perpendicular to M2. Both these locations were far from M2 and predominantly influenced only by coastal upwelling during the Southwest Monsoon. The study was based on an 18 week/fortnightly time series measurement carried out at M1, M2 and M3 spanning over a period from April to September of 2014. The physical signatures of Mud Bank, such as relatively high suspended sediments and calm sea conditions, existed at M2 by mid-June; before that, all three locations showed closely similar physical characteristics. The coastal upwelling was prevalent over the entire study area, irrespective of locations, by early-June; it was characterised by the surfacing of cool, nutrient-rich and hypoxic waters. Air temperature data spanning over the sampling period was obtained from an automated weather station (AWS) installed adjacent to the study region. At each sampling location, a portable CTD profiler with additional sensors of turbidity, PAR and chlorophyll *a* were used to record the respective high-resolution data (from every 0.2 m) up to the near-bottom waters (6 m in M1 and M2, 12 m in M3). Here, adequate caution was taken as the CTD salinity sensor can malfunction when operated in fluid muddy layers situated very close to the sea bottom^40^. For conveniently presenting the time series data and also to make the interpretation simpler and clearer, the sampling months were pooled into Pre-Monsoon (April-May), Early Monsoon (June), Peak-Monsoon (July) and Late-Monsoon (August–September) periods.

The study area is popular for fishing and frequented for such purpose; therefore, prior permission from local authorities was not required for conducting oceanographic surveys in the region. Also, the present study aimed to understand the biophysical coupling of plankton in the study area, and these organisms do not fall under any endangered/protected category. Water samples were collected from the surface (0.5 m) and sub-surface waters (5 m in M1 & M2 and 8 m in M3) using 5L Niskin samplers for measuring various physico-chemical and biological parameters. The turbidity of the surface and sub-surface waters was measured using a Eutech E120 Turbidity metre (accuracy 0.01NTU) following nephelometric principles to cross-check the CTD sensor data. Dissolved oxygen, nitrate and phosphate in the water samples were measured following standard protocols^41^. Chlorophyll *a* was concentrated by filtering 0.5 litre of water samples through Whatman GF/F filter paper and was estimated using a Trilogy Turner fluorometer as per standard procedure^42^. Zooplankton samples were collected using a standard ring net by horizontal tows just below the surface (0.5 m). The zooplankton samples were initially filtered through a 200 µm nylon sieve and the excess water in the samples were removed using a blotting paper. The biomass of the zooplankton was measured as displacement volume and the samples were preserved in 4% formalin in filtered seawater for detailed taxonomic analysis. Zooplankton sub-samples (50%) were sorted to the closest possible taxonomic group level and their abundance was estimated as per Postel *et al*.^43^. Copepods, the most abundant zooplankton, were further analysed down to the species level to assess major compositional changes during the different phases of the sampling period.

Univariate analysis was performed to compare the spatial and temporal variations in the environmental and biological parameters in the study area. Initially, the datasets were tested for their homogeneity/uniform distribution. Parametric ANOVA was used to compare the distribution of variables with homogenous distribution, whereas non-parametric ANOVA (Kruskal - Wallis ANOVA) was used to compare the distribution of variables with heterogeneous distribution. Tukeys HSD post hoc test^44^ was used to compare the pair-wise significance in parametric ANOVA while Dunn’s post hoc test helped analyse pair-wise significance in the Kruskal-Wallis test^43^. Tests of normality and parametric and nonparametric ANOVA were carried out in XL stat pro. The overall relationships between the biological and environmental variables were analysed using RDA (CANOCO 4.5). The data was initially analysed using Detrended Correspondence Analysis (DCA) to select the appropriate ordination technique. An axis gradient length <2 in DCA indicated that linear multivariate RDA was suitable for the present data^45–47^. Prior to the analysis, the biological variables were log transformed, and a partial RDA was also carried out to find out the combinations of the parameters that contributed to the response of plankton. The ordination significance was tested with Monte Carlo permutation tests (499 unrestricted permutations) (p < 0.05). Triplots were prepared to represent the results of RDA in which samples are displayed by points and environmental and biological variables by arrows.

### Data availability statement

The datasets will be published in the public data repository of CSIR-National Institute of Oceanography (CSIR-NIO). It can be accessed by anybody by giving individual request to Director CSIR-NIO or Scientist-in-charge of CSIR-NIO Regional Centre, Kochi, as per the Institutional policy.

## Electronic supplementary material

Supplementary Information

Supplementary video SV1

Supplementary video SV2
