# Supplementary material for: Mud Banks along the southwest coast of India are not too muddy for plankton
Source: Sci Rep. 2018 Feb 7;8:2544. doi: 10.1038/s41598-018-20667-9 (PMC5803250; doi:10.1038/s41598-018-20667-9)
Supplement: Supplementary file 1 — Supplementary Information [file 41598_2018_20667_MOESM1_ESM.pdf]

Manuscript Title:

Mud Banks along the southwest coast of India are not too muddy for plankton

Authors:

Jyothibabu, R., Balachandran, K.K., Jagadeesan, L., Karnan, C., Arunpandi, N., Naqvi, S.W.A., Pandiyarajan, R.S

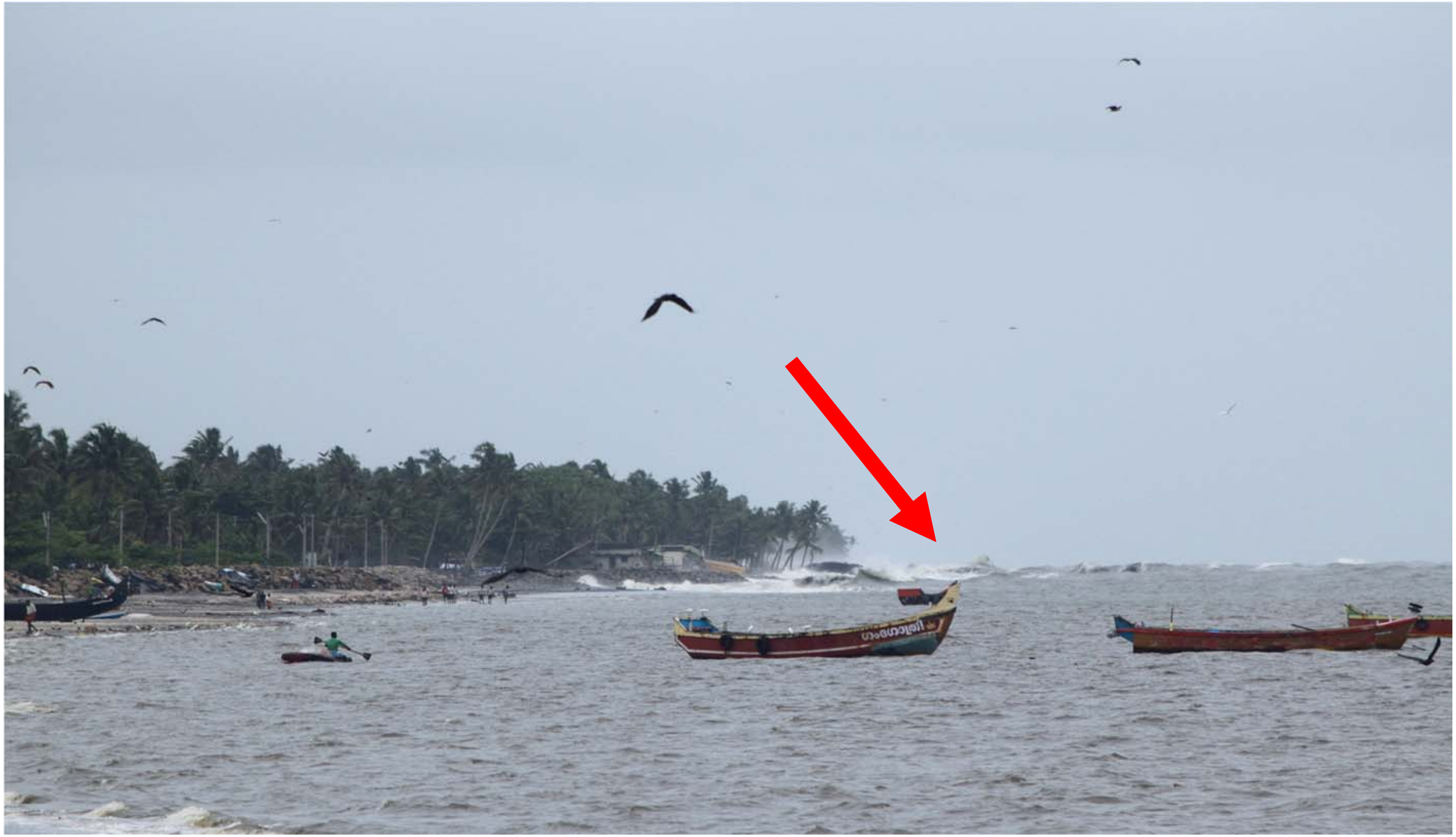

Supplementary Figure S1 - The southern boundary of the mud bank is presented, which showed a clear contrast between waves dampened calm condition in the Mud Bank and the furious sea conditions outside the Mud Bank. Wave damping is very clear in the Mud Bank. Calm sea surface condition facilitated the boats to anchor in the mud bank region. Contrasting to the mud bank, ~1km far from the mud bank is the usual monsoonal sea condition with heavy wave action indicated with a red arrow.

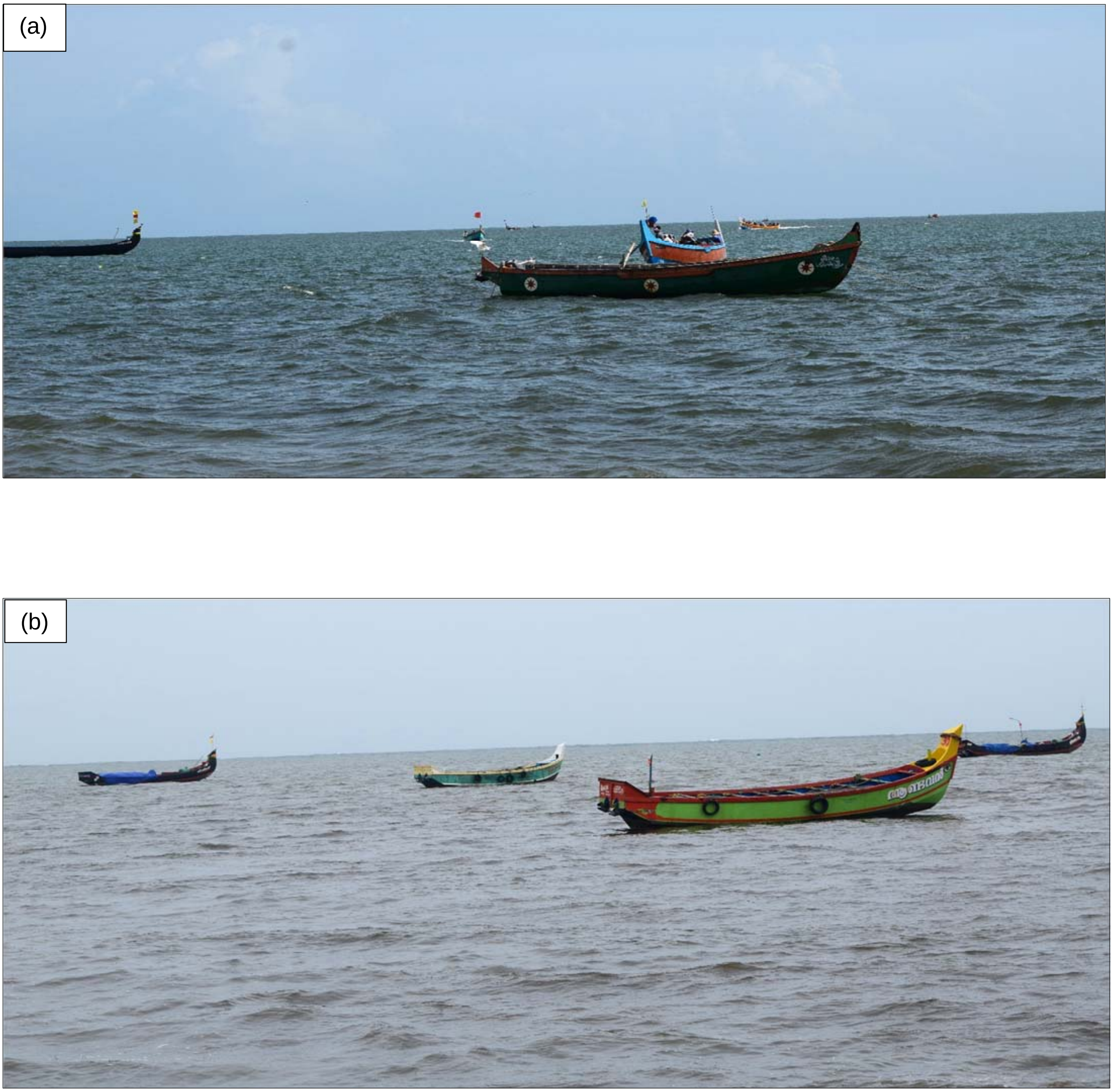

Supplementary Figure S2 - Physical appearance of mud bank during (a) clear condition and (b) turbid condition. The turbid condition is formed when significantly heavy waves attenuate at the outer boundary of Mud Bank, which occurs mainly associated with depression episodes. The high energy waves disturb the near bottom fluid muddy layer increasing the turbidity in the entire water column which usually exist for a day or two.

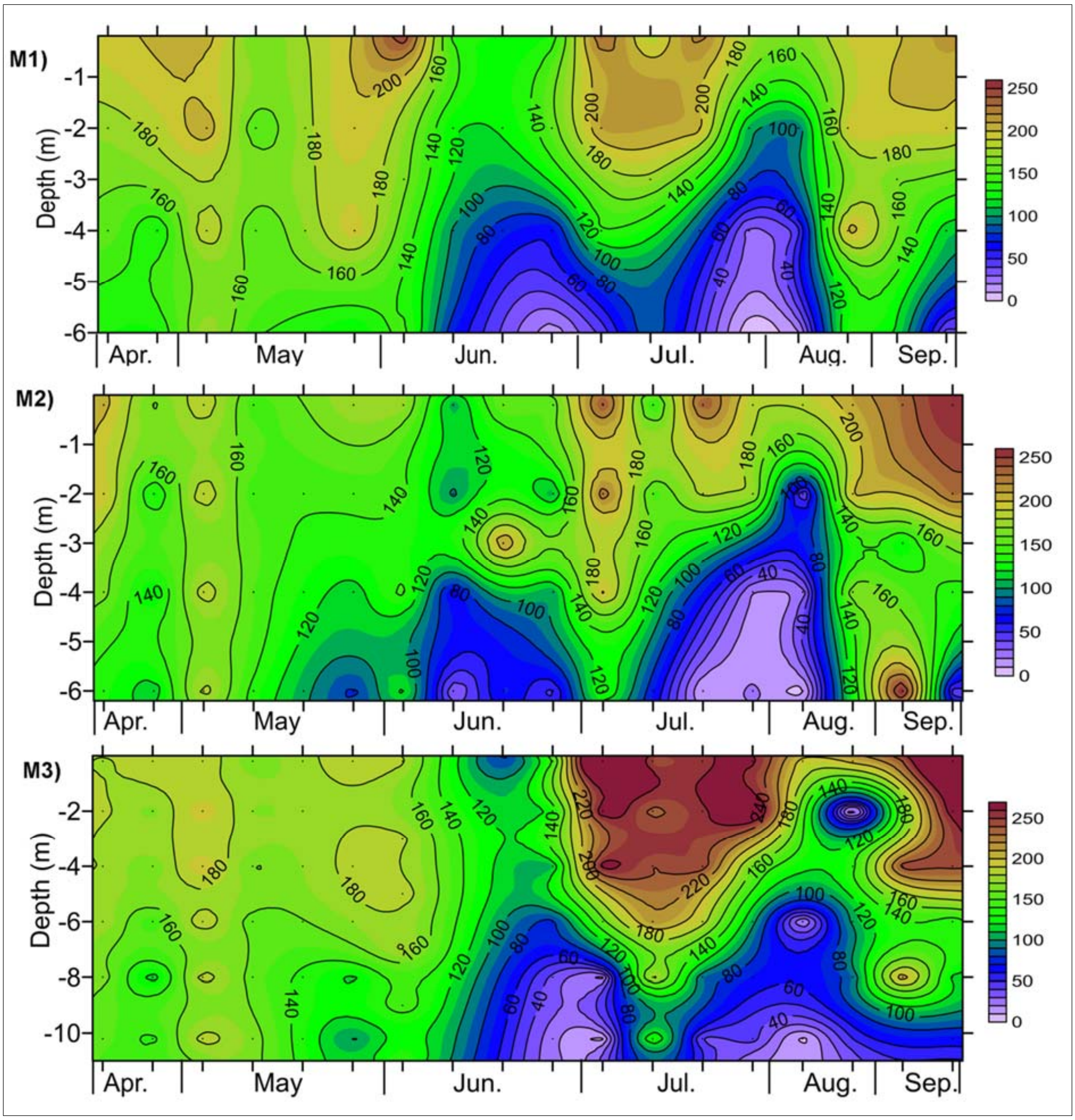

Supplementary Figure S3 - Temporal change in the vertical distribution of dissolved oxygen in M1, M2 and M3. The oxygen deficient (hypoxic waters) in the subsurface waters during the Southwest Monsoon is a clear signature of coastal upwelling in the study region

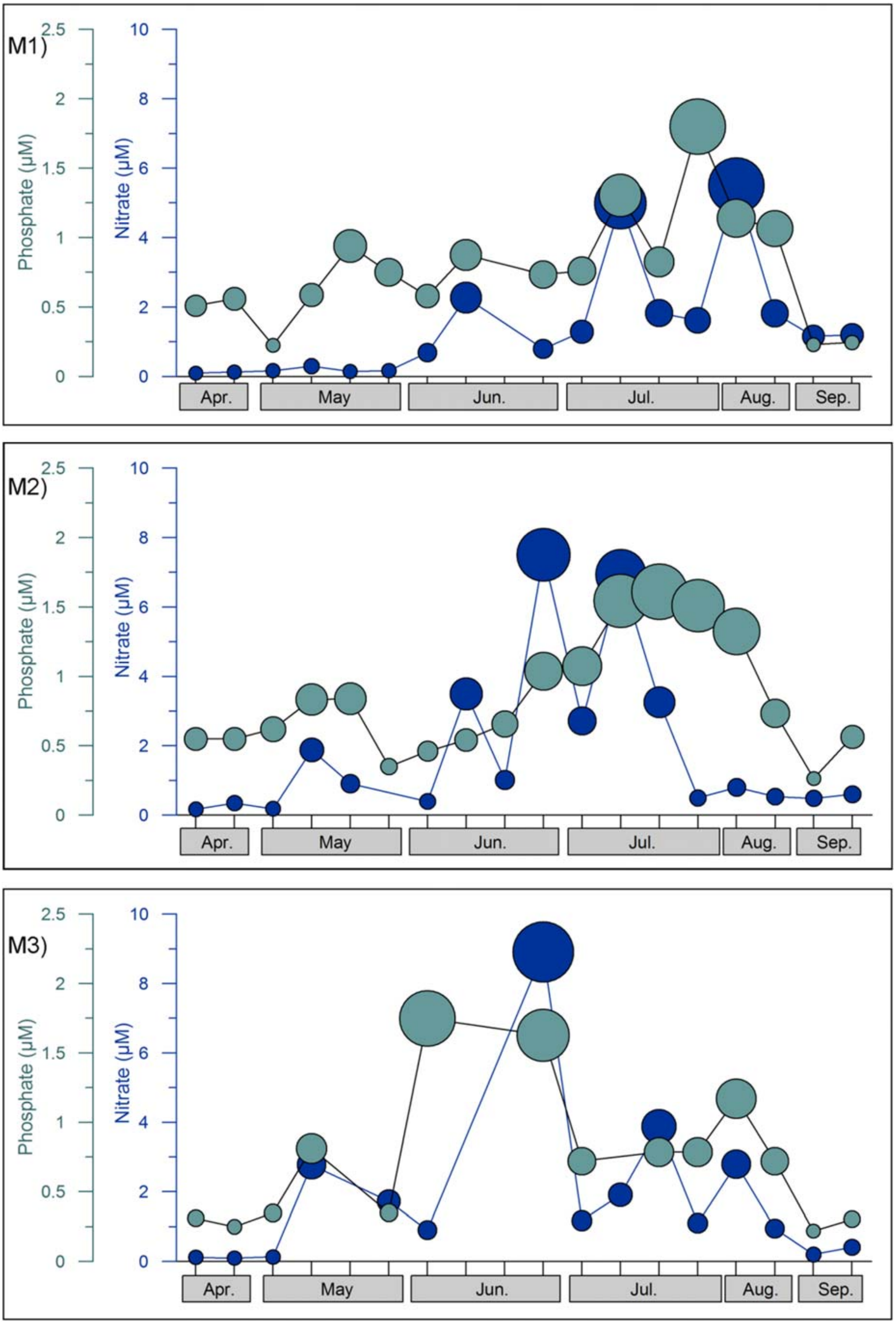

Supplementary Figure S4 - Temporal variation of nitrate (blue bubbles) and phosphate (green bubbles) at M1, M2 and M3. The bubble size represents the proportionate values of the specific parameters.

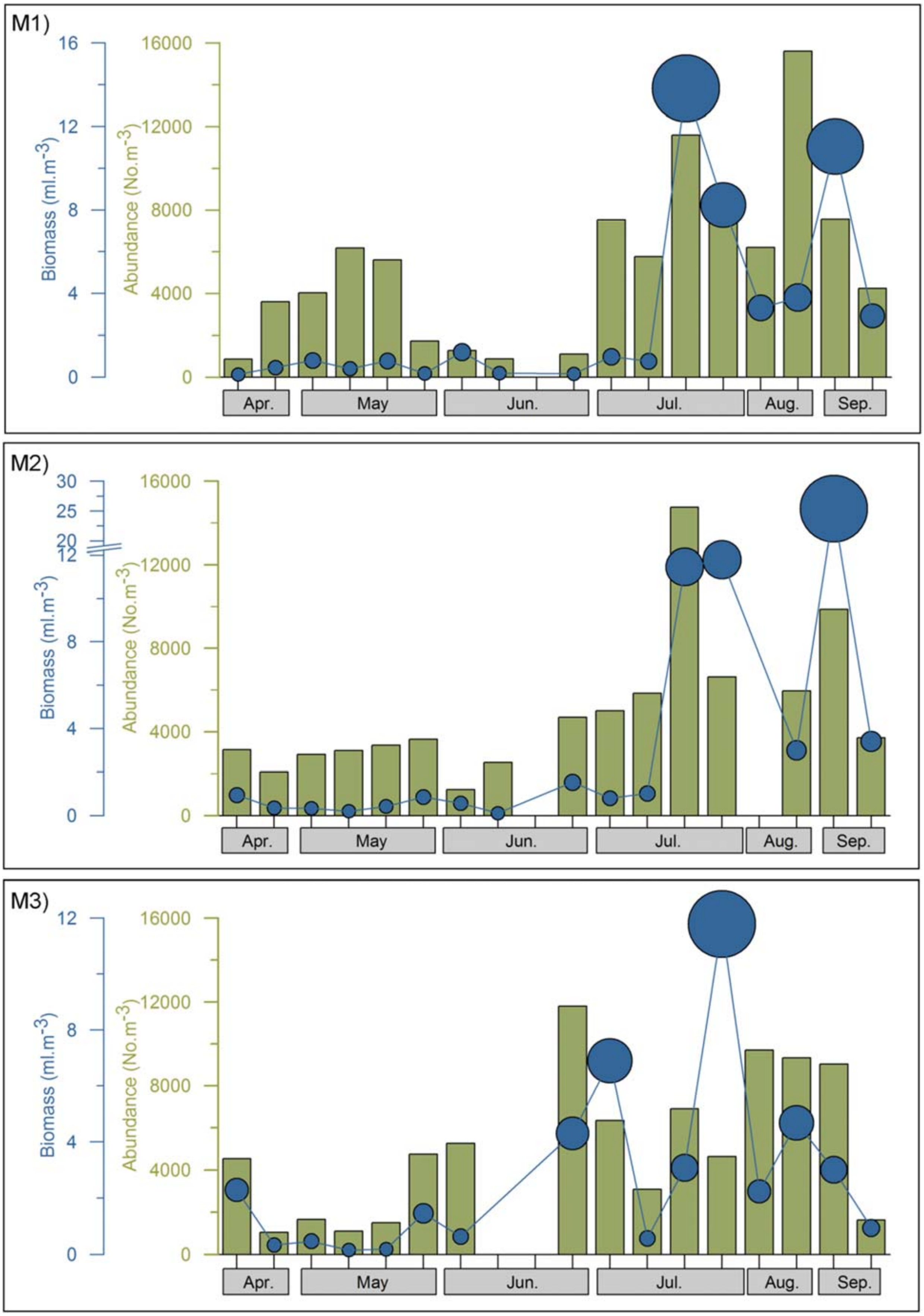

Supplementary Figure S5 - Spatial distribution of the zooplankton biomass and abundances at M1, M2 and M3. The bubble size represents the proportionate values of zooplankton biomass and abundance.

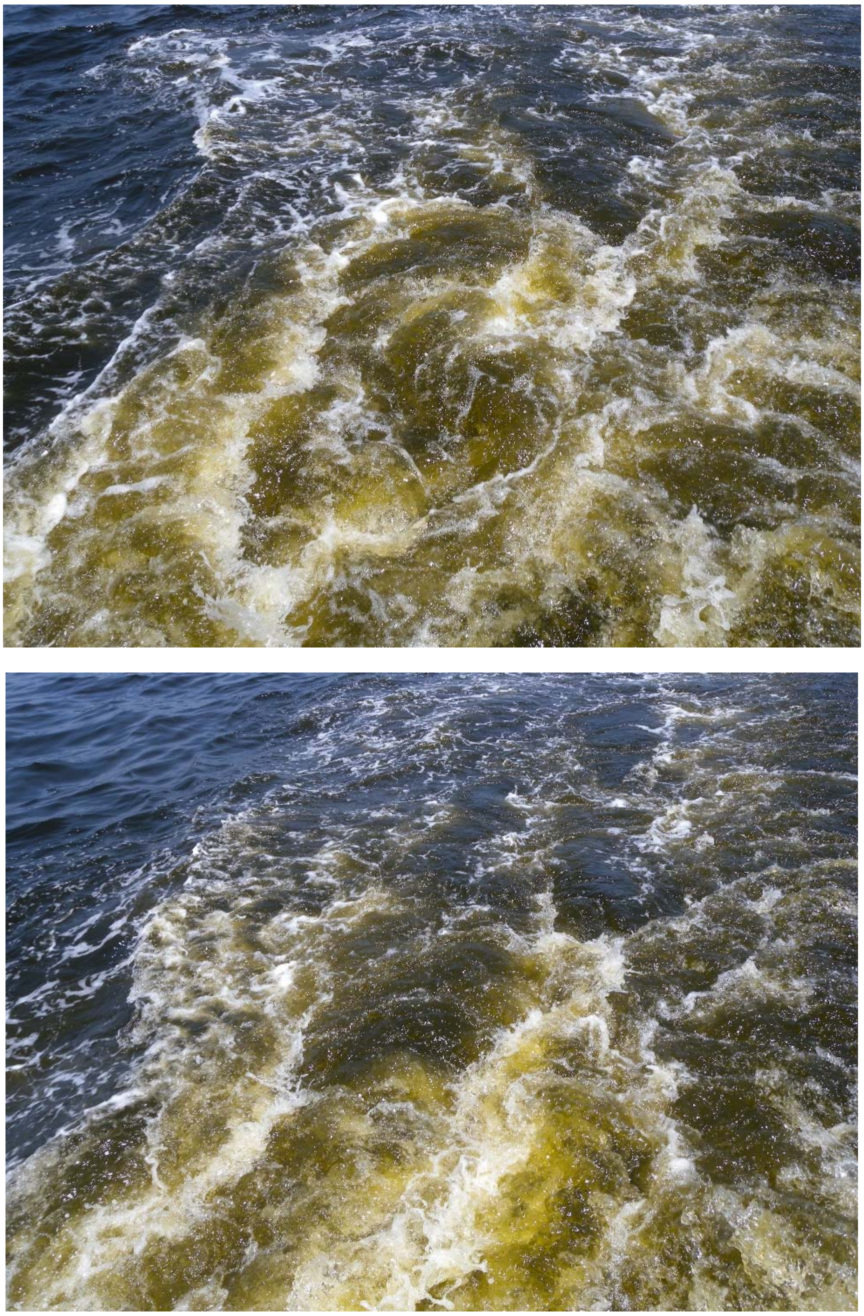

Supplementary Figure S6 - The yellowish brown discoloration evident along the trail of the sampling boat due to the blooming of long chain forming diatom *Fragilaria sp.* in the subsurface waters. This feature was widespread in the in the entire study area during the Southwest Monsoon (mud bank) period, which was a clear indication of sufficient light and nutrients available in the subsurface waters.

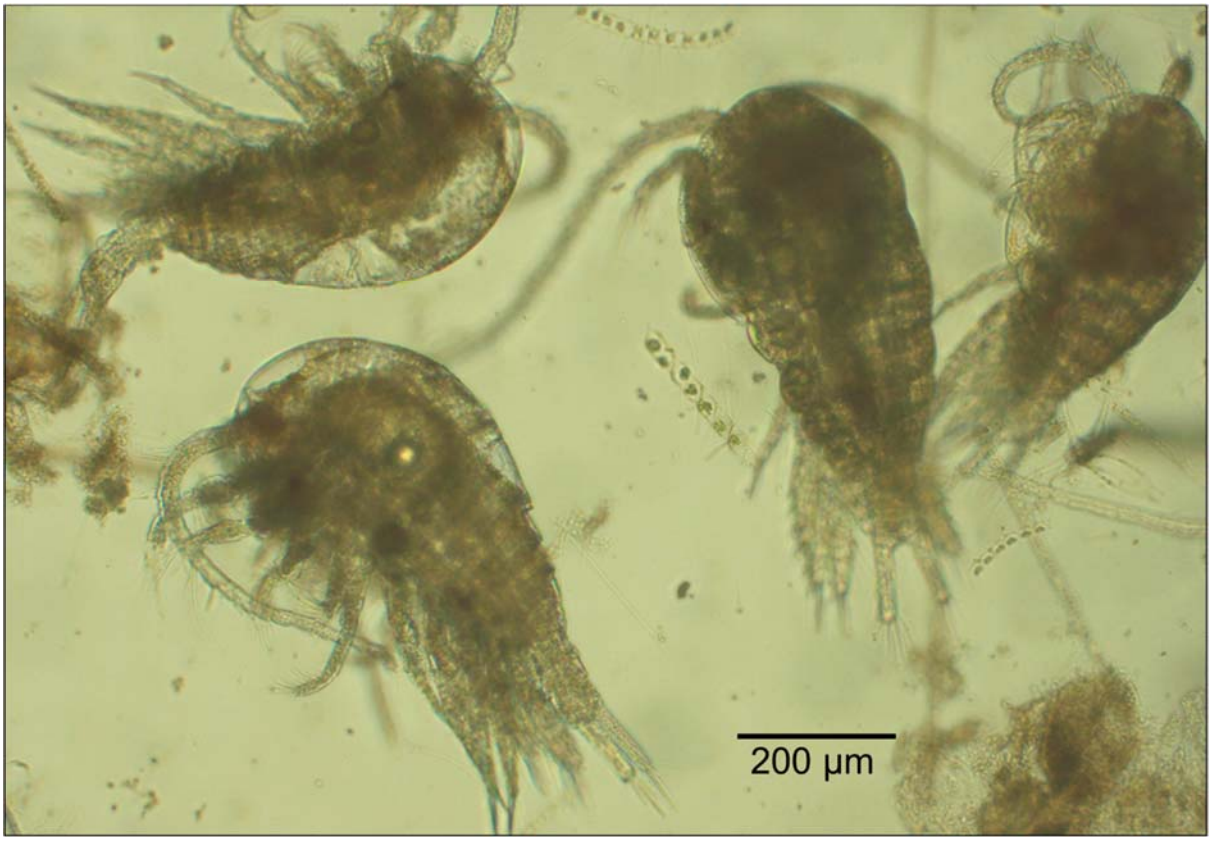

Supplementary Figure S7 - Copepod *Temora turbinata* (upwelling indicator) dominant during early Southwest Monsoon.

			Spatial variations	Temporal variations		
SI.No.	Parameters	Depth	(Between M1, M2 and M3)	M1	M2	M3
1	Salinity	Surface	NS ($p = 0.188$)	NS ($p = 0.237$)	NS ($p = 0.451$)	NS ($p = 0.294$)
		Subsurface	NS ($p = 0.210$)	NS ($p = 0.471$)	NS ($p = 0.651$)	NS ($p = 0.154$)
2	Temperature	Surface	NS ($p = 0.828$)	S ($p < 0.01$)**	S ($p < 0.01$) **	S ($p < 0.01$) **
		Subsurface	NS ($p = 0.756$)	S ($p < 0.01$) **	S ($p < 0.01$) **	S ($p < 0.01$) **
3	Dissolved Oxygen	Surface	NS ($p = 0.096$)	NS ($p = 0.887$)	NS ($p = 0.091$)	NS ($p = 0.221$)
		Subsurface	NS ($p = 0.876$)	S ($p < 0.05$)*	S ($p < 0.01$)**	S ($p < 0.01$)**
4	Turbidity	Surface	S ($p < 0.05$)*	S ($p < 0.01$) **	S ($p < 0.01$) **	S ($p < 0.05$)*
		Subsurface	NS ($p = 0.376$)	S ($p < 0.01$)**	S ($p < 0.01$)**	S ($p < 0.01$)**
5	Nitrate	Surface	NS ($p = 0.898$)	S ($p < 0.05$)*	S ($p < 0.05$)*	S ($p < 0.05$)*
		Subsurface	NS ($p = 0.093$)	S ($p < 0.05$)*	S ($p < 0.05$)*	S ($p < 0.01$)**
6	Phosphate	Surface	NS ($p = 0.693$)	S ($p < 0.05$)*	S ($p < 0.05$)*	S ($p < 0.05$)*
		Subsurface	NS ($p = 0.468$)	S ($p < 0.05$)*	S ($p < 0.05$)*	S ($p < 0.05$)*

Supplementary Table S1 - ANOVA showing the spatial and temporal variations of hydrographical parameters. Significant variations are indicated. S - Significant, NS - Not significant, * $p < 0.05$, **$p < 0.01$
